# Identification of Novel Components of the Unfolded Protein Response in Arabidopsis

**DOI:** 10.3389/fpls.2016.00650

**Published:** 2016-05-12

**Authors:** Md. Amir Hossain, Carlos Henríquez-Valencia, Marcela Gómez-Páez, Joaquín Medina, Ariel Orellana, Jesús Vicente-Carbajosa, Jan Zouhar

**Affiliations:** ^1^Centro de Biotecnología y Genómica de Plantas UPM-INIA, Universidad Politécnica de MadridMadrid, Spain; ^2^Centro de Biotecnología Vegetal, FONDAP Center for Genome Regulation, Facultad de Ciencias Biológicas, Universidad Andrés BelloSantiago, Chile

**Keywords:** Unfolded Protein Response, endoplasmic reticulum, transcription factors, abiotic stress, Arabidopsis

## Abstract

Unfavorable environmental and developmental conditions may cause disturbances in protein folding in the endoplasmic reticulum (ER) that are recognized and counteracted by components of the Unfolded Protein Response (UPR) signaling pathways. The early cellular responses include transcriptional changes to increase the folding and processing capacity of the ER. In this study, we systematically screened a collection of inducible transgenic Arabidopsis plants expressing a library of transcription factors for resistance toward UPR-inducing chemicals. We identified 23 candidate genes that may function as novel regulators of the UPR and of which only three genes (*bZIP10, TBF1*, and *NF-YB3*) were previously associated with the UPR. The putative role of identified candidate genes in the UPR signaling is supported by favorable expression patterns in both developmental and stress transcriptional analyses. We demonstrated that WRKY75 is a genuine regulator of the ER-stress cellular responses as its expression was found to be directly responding to ER stress-inducing chemicals. In addition, transgenic Arabidopsis plants expressing WRKY75 showed resistance toward salt stress, connecting abiotic and ER-stress responses.

## Introduction

Eukaryotes maintain homeostasis in the endoplasmic reticulum (ER) through a set of signaling pathways known as the Unfolded Protein Response (UPR; Walter and Ron, 2011). As a consequence, the stress conditions caused by accumulation of unfolded proteins in the ER lumen are alleviated by changes in the expression of ER-resident chaperones, folding enzymes, and other components involved in the ER quality control and the ER-associated degradation systems. Functionally, all eukaryotic branches rely on ER membrane-localized sensors of misfolded luminal proteins.

In yeast, Ire1p is a single pass ER membrane protein that poses both kinase and endoribonuclease activities. Under conditions of ER-stress, the Ire1p catalyzes the splicing of *HAC1* mRNA in the cytosol (Sidrauski and Walter, 1997). The spliced form encodes a basic leucine zipper (bZIP) transcription factor Hac1p able to upregulate the ER-stress response genes (Mori et al., 1996).

In metazoans, the evolutionary conserved branch is represented by two Ire1p-homologs, IRE1α and IRE1β that show certain degree of specialization (Tirasophon et al., 1998; Wang et al., 1998; Bertolotti et al., 2001). Similarly to yeast, the activated IRE1α/β process *XBP-1* (X-box Binding Protein) mRNA and the translated product XBP-1 controls expression of ER-localized chaperones and degradation-related proteins (Yoshida et al., 2001). In addition, metazoans contain two additional branches of the UPR. Two ER membrane-localized Activating Transcription Factors 6 (ATF6α and ATF6β) represent the second arm. After sensing unfolded protein accumulation in the ER lumen, the ATF6 proteins are translocated to the Golgi apparatus where they undergo proteolytic activation (Haze et al., 1999). The resulting bZIP-like transcription factors upregulate the ER-stress response genes. The third branch is the evolutionary youngest and is primarily responsible for translation attenuation via the PKR-like Endoplasmic Reticulum eIF2α Kinase (PERK). The PERK activation results in the decrease of the overall levels of protein translation, thus allowing the ER to process the existing load of unfolded proteins (Harding et al., 1999).

Plants show clear evolutionary conservation of the IRE1 and the ATF6 pathways (Howell, 2013). Consistently with metazoans, Arabidopsis contains two functional homologs of IRE1, IRE1a, and IRE1b (Koizumi et al., 2001). Both IRE1a and IRE1b endoribonuclease activities target *bZIP60* mRNA, a homolog of mammalian *XBP-1*. Though IRE1 is an otherwise conventional protein kinase, its only known phosphorylation substrate is IRE1 itself. In mammalian IRE1, phosphorylation within the kinase activation loop significantly increases endoribonuclease activity (Prischi et al., 2014). Interestingly, in both mammals and plants, it is the kinase domain and not the *bZIP60/XBP1* ribonuclease activity that is required for the IRE1-mediated autophagy, suggesting that IRE1 can indeed phosphorylate other substrates (Ogata et al., 2006; Liu et al., 2012). The mammalian ATF6 has three homologs in Arabidopsis, bZIP17, bZIP28, and bZIP49. Two of them, bZIP17 and bZIP28 were further characterized. Under ER stress conditions, both transcription factors translocate from the ER to the Golgi (Che et al., 2010). In eukaryotes, the secretory cargo is delivered from the ER to the Golgi apparatus via COPII vesicles. Whether both bZIP17 and BZIP28 use this molecular mechanism is currently unknown. However, the recently described interaction between bZIP28 and small GTPase Sar1 strongly suggests that both bZIP17 and bZIP28 may use the conventional COPII trafficking pathway (Srivastava et al., 2012). At the Golgi apparatus, bZIP17 and bZIP28 are proteolytically cleaved by site-2 protease and released to translocate into the nucleus to activate ER stress response gene expression (Che et al., 2010).

In higher plants, massive amounts of storage proteins are synthesized during seed maturation generating demands for an efficient protein folding in the ER. Therefore, it is not surprising that the first evidence of the ER stress in plants came from studies of mutants in seed storage protein genes in maize (Boston et al., 1991). In addition, increased protein synthesis during responses to adverse abiotic (salt, heat, and drought) and biotic stresses can also negatively affect folding capacity of the ER. In Arabidopsis, IRE1-mediated splicing of *bZIP60* and the activation of bZIP17 and bZIP28 were identified as the outcomes of the heat stress response (Gao et al., 2008; Che et al., 2010; Deng et al., 2011). Even though the cellular functions of both IRE1 homologs largely overlap, in stress responses they exhibit certain preference, as the IRE1b is mainly responsible for the abiotic stress signaling, while the IRE1a appears to play principal role in biotic stress responses (Moreno et al., 2012). Similarly, salt stress triggers proteolytic processing of bZIP17 but not bZIP28, suggesting an intriguing layer of specificity within the ATF6-homolog family of proteins (Liu et al., 2007a,b).

Recently, a heterotrimeric NF-Y complex was identified as a nuclear interacting partner of bZIP28 (Liu and Howell, 2010). The resulting transcriptional complex then consists of ER stress-specific homo- or heterodimers of bZIP28 and a general transcription factor NF-Y and binds to consensus promoter sequence corresponding to the ER stress-responsive element I (Liu and Howell, 2010). In addition, 12 transcription factors were found to be more than 3-fold upregulated under chemically induced ER stress conditions (Iwata et al., 2008). Out of them, three NAC transcription factors, NAC062, NAC089, and NAC103, are dependent on bZIP60 (Iwata et al., 2008) and were further characterized. NAC062 and NAC089 are membrane localized TFs, which under ER stress relocate to the nucleus (Yang et al., 2014a,b). In contrast, NAC103 does not have a predicted transmembrane domain and it is localized to the cytoplasm (Sun et al., 2013). While NAC062 and NAC103 relay ER stress transcriptional signals to ensure cell survival (Sun et al., 2013; Yang et al., 2014a), NAC089 regulates ER stress-induced programmed cell death (Yang et al., 2014b). These findings suggested that additional effectors might further amplify or tune the unfolded protein response. These proteins may then either physically interacts with the known UPR signaling components (e.g., NF-Y complex), function as their downstream targets (e.g., NAC062, NAC089, and NAC103) or both.

We decided to systematically analyze a contribution of individual transcription factors to the ER stress responses. In this work, we screened a collection of inducible transgenic plants expressing a library of Arabidopsis transcription factors for resistance toward dithiothreitol (DTT), a well-known ER stress-inducing agent. The identified candidate transgenic lines were verified in a follow-up assay using tunicamycin as an independent ER stress-inducing drug. Most of the identified candidates represent genes with potentially novel roles in the Unfolded Protein Response. Subsequently, we analyzed several candidate genes for molecular phenotypes under ER-stress conditions.

## Materials and methods

### Plant material

For the ER-stress resistance screen, a collection of Arabidopsis plants expressing a library of transcription factors under an inducible promoter in the pER8 vector was utilized. The conditional expression may be of advantage in cases of potential deleterious effects on the development of stably transformed plants (Coego et al., 2014). This collection was generated within the TRANSPLANTA (TPT) consortium (Coego et al., 2014). All analyzed lines were T3 homozygotes. To analyze a candidate gene expression in various genetic backgrounds, we used previously identified and characterized T-DNA mutants in *bZIP17* (SALK_104326; Liu et al., 2007b), *bZIP28* (SALK_123659; Liu et al., 2007a), *bZIP60* (SAIL_283_B03; Deng et al., 2011), and the *ire1a ire1b* double mutant (SALK_018112, SAIL_238_F07; Moreno et al., 2012), here referred to as *ire1*.

### Primary screen for a DTT resistance

Ten seeds of each transgenic line were surface sterilized with sodium hypochlorite, thoroughly washed and stored in sterile water for 3 days at 4°C to eliminate dormancy and ensure uniform germination. Subsequently they were sowed on half-strength Murashige and Skoog media (1/2 MS) agar (0.7% w/v) plates supplemented with 10 μM estradiol and 3.5 mM DTT and germinated at 22°C with a 16 h photoperiod (8 h dark) under fluorescent illumination. The seedlings were analyzed at 4 and 7 days after sowing for presence of green cotyledons.

### Candidate verification assays

The phenotypes of identified candidates were verified using tunicamycin (Sigma) as an independent ER stress-inducing drug. Seeds were germinated in 1/2 MS liquid media supplemented with 10 μM estradiol and 1.2 μg/mL of tunicamycin. Germinated seedlings were analyzed for growth vigor and presence of green cotyledons.

### Bioinformatics

For Arabidopsis developmental series, we utilized the AtGenExpress resources (Schmid et al., 2005). For biotic and abiotic stress series, we used the AtGenExpress transcriptomic data available for shoot tissue at the Botany Array Resource (Toufighi et al., 2005). The experimental data and conditions were previously described (Kilian et al., 2007) and are available under the following identification codes: cold 1 and 24 h (AtGenExpress_Stress_1), osmotic 1 and 24 h (AtGenExpress_Stress_2), salt 1 and 24 h (AtGenExpress_Stress_3), drought 1 and 24 h (AtGenExpress_Stress_4), heat 1 and 24 h (AtGenExpress_Stress_9) and *Pseudomonas syringae* inoculation 4 and 24 h (ATGE_ExpID_168). To visualize both developmental and stress data, color-coded Clustered Image Maps were generated using the CIMminer online tool (http://discover.nci.nih.gov/cimminer/).

An undirected gene co-expression network was constructed using publicly available ATTED-II database tools (Obayashi et al., 2009), using default conditions. The ATTED-II database uses Mutual Rank (MR) as a measure of gene co-expression, which is visualized in the graphical output by three types of edge thickness. The bold edges represent MR < 5, normal edges have 5 ≤ MR < 30 and thin edges represent MR ≥ 30 (Obayashi et al., 2009). We chose to restrict the protein-protein interaction data to reduce a possible bias toward better-characterized genes. The co-expression network was constructed using candidate genes and five known UPR signaling components (*bZIP17, bZIP28, bZIP60, IRE1a, IRE1b*) as seeds. Due to the lack of co-expression data, four candidate genes were not included in the analysis (At1g50680, At1g51120, At4g36990, and At5g43290). The resulting co-expression network layout was created in the Cytoscape framework environment (Shannon et al., 2003). The ATTED-II database tools were also used to identify gene ontology terms within the datasets.

### Quantitative RT-PCR analyses

For quantitative RT-PCR analyses, the seedlings were grown for 1 week on 1/2 MS agar (0.7% w/v) and subsequently transferred for the indicated period of time into liquid 1/2 MS supplemented with indicated concentrations of estradiol and/or DTT. Total RNA was isolated using TRIzol reagent (Invitrogen, Life Technologies) according to manufacturer's protocol and treated with DNAse I (Fermentas, Thermo Scientific). For cDNA synthesis, the SuperScript II first-strand RT-PCR kit (Invitrogen, Life Technologies) was utilized. Arabidopsis *UBIQUITIN* (At5g25760) mRNA level was used as a control. Oligonucleotides used for PCR amplifications are listed in Supplemental Table 1.

### Salt stress and chlorophyll quantification assay

Four days old seedlings grown in 1/2 MS liquid medium in the presence of 10 μM estradiol were transferred into same medium containing 125 mM NaCl for 30 h. Chlorophyll (a + b) concentrations were quantified by spectroscopy in 100% methanol extracts according to previously reported methods (Porra et al., 1989).

### Protein detection and antibodies

Total protein samples were prepared from seedlings homogenized in liquid nitrogen and extracted in 2x Laemmli buffer (Bio-Rad). Gel loading was normalized to the seedling fresh weight. The anti-BiP (at-95, Santa Cruz Biotechnology) and anti-SYP2 (da Silva Conceicao et al., 1997) primary antibodies were used in immunoblots at 1:1000. The goat anti-rabbit IgG-HRP (sc-2030, Santa Cruz Biotechnology) secondary antibody was used at 1:5000. Detection was performed using the Amersham ECL Plus reagent.

## Results and discussion

### Setting up the screening conditions

**Figure 1:**
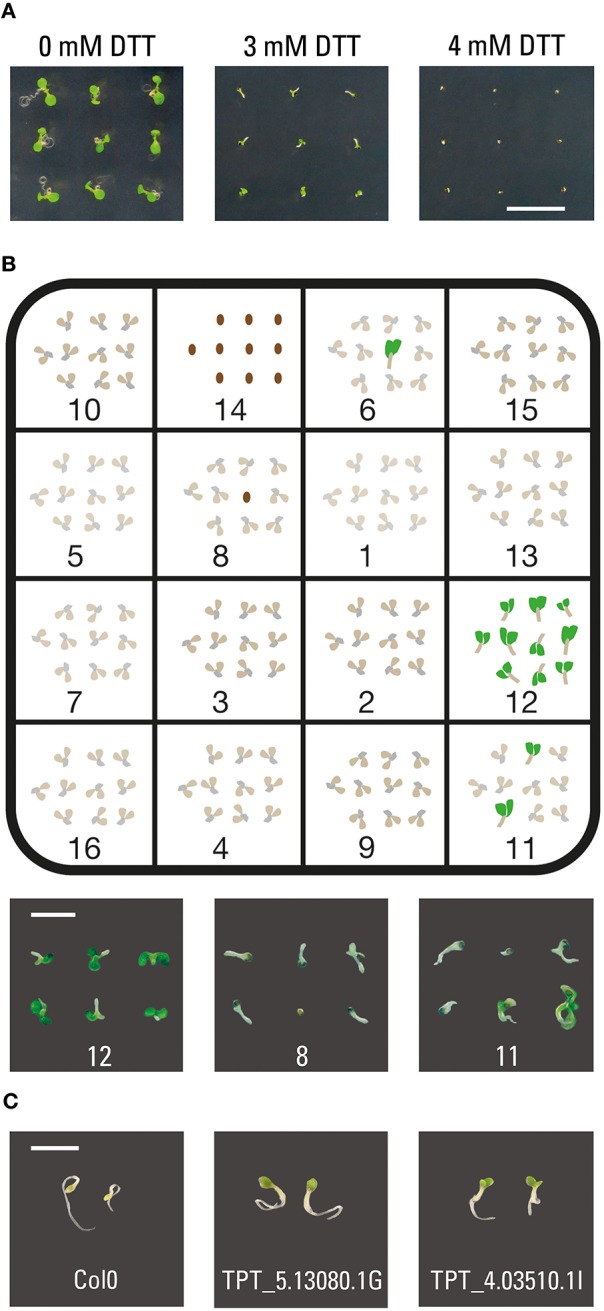
**Screening for resistance toward an ER stress-inducing drug. (A)** Arabidopsis Col0 sensitivity toward DTT. Col0 seeds were sowed on MS agar supplemented with 10 μM estradiol and 0, 3, or 4 mM DTT and grown for 4 days. **(B)** Representation of a typical screening plate. TPT lines were randomly distributed and 10 seeds of each genotype sowed on 1/2 MS agar supplemented with 10 μM estradiol and 3.5 mM DTT. Line 12 represents a candidate line, line 8 shows a typical phenotype of wild-type plants or sensitive TPT lines and line 11 is an example of an undesired phenotype variation. **(C)** Examples of the verification assay. Seedlings were grown in liquid 1/2 MS supplemented with 10 μM estradiol and 1.2 mg/L tunicamycin. Scale bar, 10 mm **(A)**, 3 mm **(B)**, 4 mm **(C)**.

Before analyzing the inducible lines expressing individual transcription factors from the TRANSPLANTA collection, we evaluated the sublethal concentration of the UPR inducing drug DTT on Arabidopsis wild-type plants. In 3 mM DTT the wild-type seedlings developed green cotyledons, while in 4 mM DTT the seed coats opened but the seedling development was severely arrested (Figure 1A). Therefore, a concentration of 3.5 mM DTT was chosen for the subsequent chemical screening. Under these conditions, the wild-type seedlings and non-resistant TPT lines would initiate germination but fail to develop green cotyledons and eventually die (Figure 1B).

### Forward chemical genomics screen to identify resistance toward DTT, an ER stress-inducing drug

Using 1/2 MS agar plates supplemented with 10 μM estradiol and 3.5 mM DTT, we analyzed 755 individual TPT lines that represent 405 unique genes. The majority of the transgenic lines mimicked the wild-type phenotype (Figure 1B, example 8). The transgenic line was considered a candidate when at least 90% of the germinated seedlings exhibited green cotyledons on the 4th day after seed sowing (Figure 1B, example 12). We identified 23 candidate genes that showed such phenotype (Table 1). Importantly, majority of the candidate genes demonstrated resistance in more than one transgenic T3 line (Table 1). However, three candidate genes (At1g51120, MYB27, and HSF4) were represented in the collection by only one homozygous T3 line. Transgenic lines where only some seedlings had green cotyledons were not considered as candidates (Figure 1B, example 11). Eight genes were represented by transgenic lines, which displayed green cotyledons on the 7th day after seed sowing. These candidates were of particular interest and are marked in Table 1 in bold.

**Table 1:** **Candidate genes identified in the screen**.

**TF family**	**AGI code**	**TPT lines**	**Gene name**
bZIP	**At1g08320**	F, G, I	**bZIP21**
	**At2g04038**	C, F	**bZIP48**
	At4g02640	D, E	bZIP10
WRKY	**At5g13080**	G, H	**WRKY75**
	**At5g43290**	A, D	**WRKY49**
	At5g49520	A, B	WRKY48
RAV	At1g50680	B, D	n/a
	At1g51120	F	n/a
	At3g25730	A, G	EDF3
MYB	**At3g13540**	B, D, F	**MYB5**
	At3g53200	F	MYB27
	At3g55730	A, F	MYB109
DOF	At5g60200	A, E	TMO6
	**At5g65590**	F, I	**SCAP1**
G2-like	**At2g01060**	A, F, G	**MYB-L**
RING-H2	**At4g03510**	H, I	**RMA1**
AP2/EREBP	At1g77200	C, H	DREB A-4
CCHC	At2g28910	C, D	CXIP4
bHLH	At3g06120	C, E	MUTE
CCAAT-HAP3	At4g14540	A, C	NF-YB3
HSF	At4g36990	D	HSF4/TBF1
MADS	At5g06500	B, G	AGL96
NAC	At5g46590	D, G	ANAC096

*The gene affiliation with the TF families as defined in the AGRIS database (http://arabidopsis.med.ohio-state.edu/AtTFDB). Candidates that displayed resistance toward DTT on the 7th day after sowing are highlighted in bold. The identified resistant transgenic T3 lines are indicated in A to I capital letters, corresponding to the TRANSPLANTA identification codes (Coego et al., 2014). The T3 lines used in the tunicamycin verification assay are underlined*.

### Candidate verification assay

While DTT triggers UPR through disruption of the correct formation of disulfide bonds in the endoplasmic reticulum, tunicamycin disrupts correct protein N-glycosylation, which results in the unfolded protein accumulation (Howell, 2013). We selected one transgenic line for each gene candidate (Table 1) and subjected seedlings of these inducible lines to tunicamycin in the verification assay. All 23 TPT lines presented resistant phenotypes comparing to the Col0 plants. Examples of the observed phenotypes are shown in Figure 1C.

### Tissue expression patterns of candidate genes

**Figure 2:**
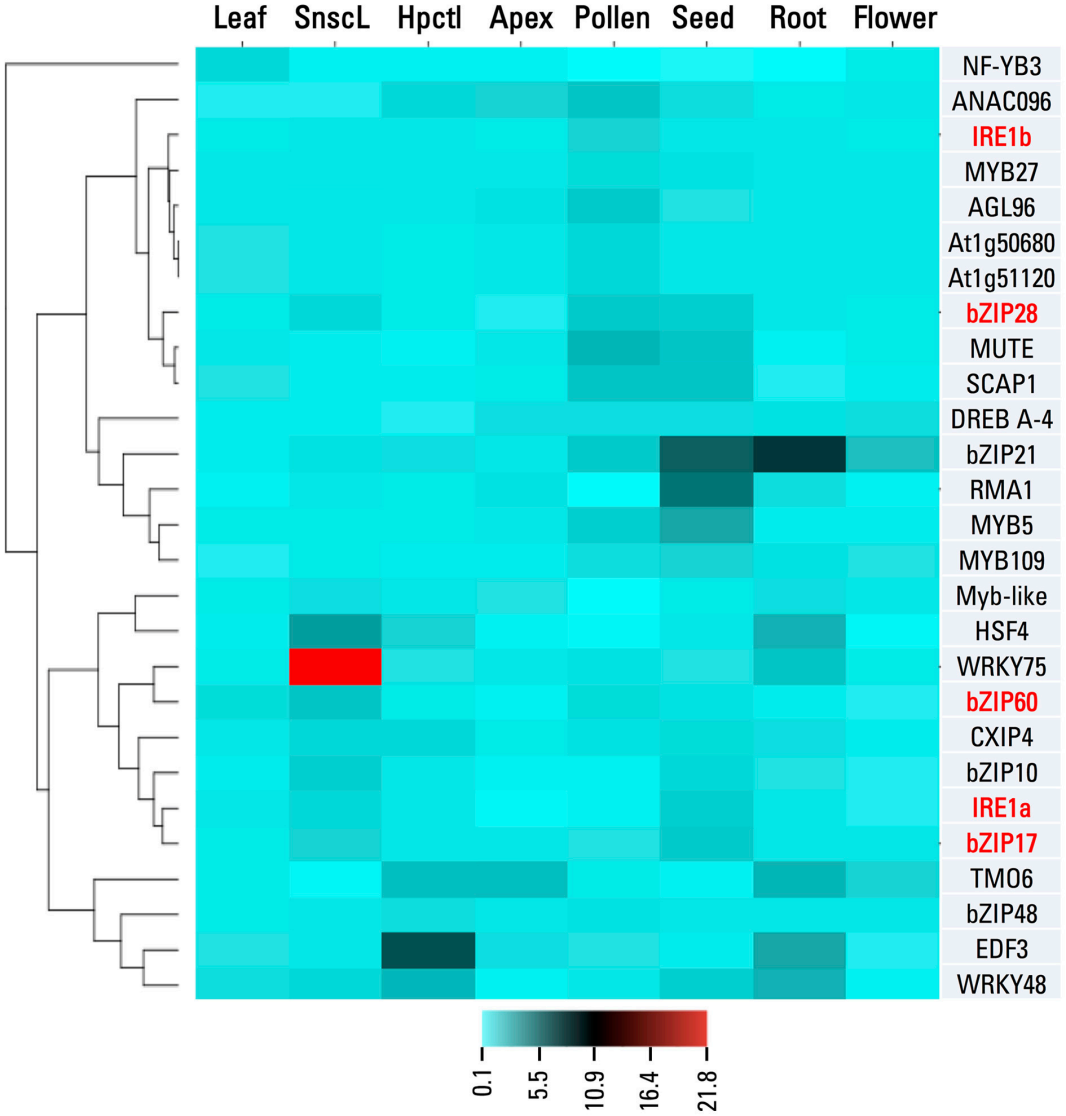
**One-matrix Clustered Image Map analysis of candidate genes and the known UPR effectors in various organs and tissues**. Leaf, rosette leaf; Hpctl, hypocotyl; Flower, flower stage 1 ; Apex, vegetative shoot apex; Seed, seeds stage 10 without siliques; Pollen, mature pollen; SnscL, senescing leaf. Gene expression levels of selected candidate genes and known UPR signaling components (red-typed names) were obtained from the Botany Array Resource database. Both genes and conditions were clustered using correlation as distance method. Color-coded scale indicates normalized expression levels.

In order to characterize the candidate genes and to identify similarities to previously identified UPR components, we analyzed their tissue expression patterns across various developmental stages using publicly available bioinformatics tools (Winter et al., 2007). Such analysis may suggest involvement of a candidate gene in particular physiological processes. In case of genes previously associated with the UPR pathways, we observed five major tissue expression patterns. The bZIP17, bZIP28, and IRE1a show high expression in the late stages of seed development, consistently with the high seed storage protein expression during the seed filling stage. As seed storage proteins pass through the endomembrane system, the ER-localized chaperones and UPR sensing machinery likely play an important role in the seed filling homeostasis (Hatano et al., 1997). Of 23 gene candidates, six genes (*bZIP21, RMA1, MYB5, WRKY48, SCAP1*, and *MUTE*) show prominent expression in the developing embryos (Figure 2). The bZIP28 and IRE1b are strongly expressed in pollen and bZIP60 is expressed principally in senescing tissues. The components of the UPR pathways were previously associated with male and female gametophyte development and pollen elongation (Koiwa et al., 2003; Yang et al., 2009; Conger et al., 2011). Of our candidate genes, five showed conspicuous flower or pollen expression patterns (Figure 2). A double mutant of *IRE1* in Arabidopsis shows defects in root cell elongation, a process characterized by rapid secretory pathway-dependent synthesis of cell wall material (Chen and Brandizzi, 2012). Consistently with these findings, six candidate genes (*bZIP21, HSF4, WRKY75, TMO6, EDF3*, and *WRKY48*) exhibit root expression patterns (Figure 2). Interestingly, several of these genes show also high expression in the hypocotyl, a plant organ with a similar high activity of vesicular trafficking within the endomembrane system. The correct ER-folding processes are also crucial for meristem development and maintenance (Ishiguro et al., 2002). Arabidopsis mutant in the *SHEPHERD* gene shows expanded floral and apical meristems, suggesting that the corresponding protein likely acts as the ER-localized chaperone of CLAVATA proteins. Among our candidates, we identified two genes (*TMO6* and *ANAC096*) with notable floral meristem expression patterns. In summary, the candidate genes show clear expression patterns in tissues with a high protein synthesis and a high activity of the endomembrane system, supporting their putative role in the ER homeostasis regulation.

### Gene co-expression network analyses

**Figure 3:**
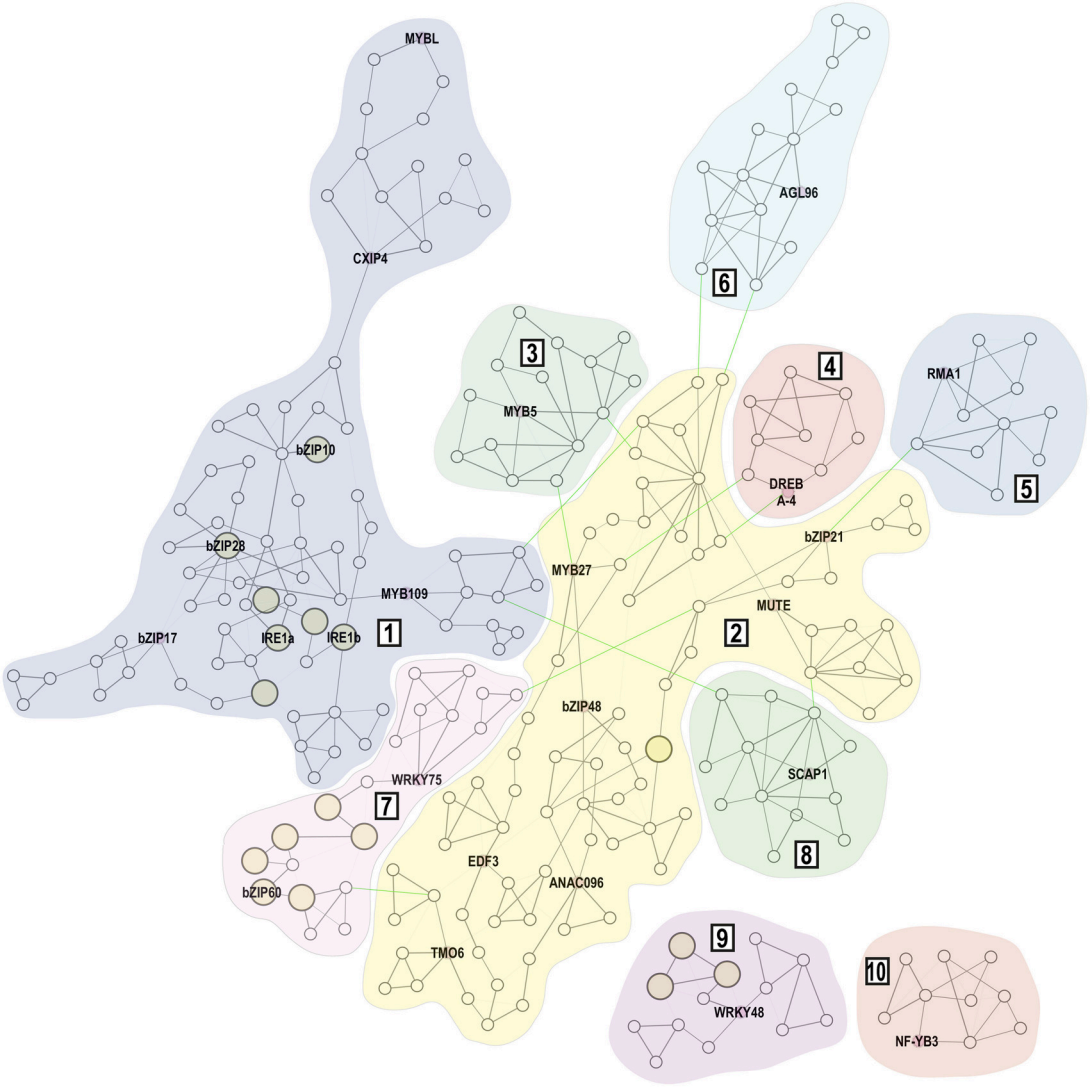
**Gene co-expression network of 23 candidate genes and the known UPR signaling machinery**. Gene co-expression network contains 265 nodes connected by 462 edges. Expression modules are indicated by Arabic numerals (1–10). Within each module, the MR < 5 is represented by bold edges, while normal edges have 5 ≤ MR < 30 and thin edges represent MR ≥ 30. Edges that connect individual expression modules but represent co-expression with lower correlation (MR ≥ 30) are highlighted in green. Nodes that are marked by larger yellow circles are genes annotated with the Endoplasmic Reticulum Unfolded Protein Response (GO:0030968) ontology term.

To analyze possible genetic interactions within our dataset, we constructed a gene co-expression network using both genes identified in our screening and the known UPR-signaling components. Four genes of our dataset were not included in the co-expression analysis. Due to the lack of a corresponding probe within the Affymetrix Arabidopsis ATH1 genome array, *WRKY49* is not represented in the Arabidopsis transcriptomics database. In addition, two members of the AP2/B3 family (At1g50680 and At1g51120) and *HSF4/TBF1* have no co-expression data available due to cross-hybridization. The resulting network comprises 265 nodes (genes) and 462 edges, representing gene-to-gene co-expression (Figure 3; Supplemental Table 2). We considered a group of co-expressed genes as a co-expression module when edges within this group showed MR < 30. The network then contains two larger modules (1 and 2) with 79 and 80 nodes, respectively. Module 1 includes *bZIP17, bZIP28* and both *IRE1* homologs, which encode known UPR signaling components. Six modules (3–8) were found loosely connected by edges with low co-expression measures (MR ≥ 30) to both larger modules, while two modules (9 and 10) were not connected by any edges to other modules.

Subsequently, we analyzed all nodes of the co-expression network for relevant gene ontology terms. Seventeen nodes were annotated with the Endoplasmic Reticulum Unfolded Protein Response (ER-UPR, GO:0030968) ontology term (Supplemental Table 3). Within our 23 candidates, the bZIP10 and HSF4/TBF1 transcription factors share this GO annotation. The bZIP10 TF belongs to the group C of the *bZIP* gene family in Arabidopsis and physically and functionally interacts with bZIP53 and ABI3 transcription factors (Alonso et al., 2009). The resulting heterotrimeric protein complex controls expression of seed maturation genes, further strengthening the role of the UPR machinery in seed filling regulation (Alonso et al., 2009). The HSF4/TBF1 transcription factor has been previously associated with the UPR as the corresponding *tbf1* mutant shows sensitivity toward the UPR inducer tunicamycin (Pajerowska-Mukhtar et al., 2012). In addition, HSF4 binds directly to the *cis* elements within the BiP2 promoter resulting in severely compromised BiP2 induction in the *tbf1* mutants upon salicylic acid treatment when compared to the wild-type plants (Pajerowska-Mukhtar et al., 2012). However, the direct involvement of HSF4 in the UPR processes has been recently challenged and it is possible that the observed phenotypes are due to an indirect participation of HSF4/TBF1 in the UPR (Nagashima et al., 2014).

Within the *WRKY48* cluster we identified three genes with the ER-UPR gene ontology annotation, pointing to the hypothetical role of WRKY48 in the UPR processes. The remaining 13 genes from the ER-UPR GO category are co-expressed with the known UPR signaling machinery (Supplemental Table 2). Interestingly, nearly half of these genes form the *WRKY75/bZIP60* co-expression cluster.

In Arabidopsis, the NUCLEAR FACTOR Y, SUBUNIT B3 (NF-YB3), a candidate identified within an isolated expression cluster in our co-expression analysis, belongs to a gene family of 13 members (Siefers et al., 2009). However, only NF-YB3 was previously identified as an interacting partner of bZIP28 in plants treated with ER stress-inducing drugs (Liu and Howell, 2010). NF-YB3 is localized to the cytoplasm but it is recruited into a nuclear-localized NF-Y heterotrimeric complex by NF-YC2, which is actively upregulated by tunicamycin and during seed development (Liu and Howell, 2010). It was hypothesized that the NF-Y trimer interaction with bZIP28 might be important in maintaining or adding to the magnitude of the UPR (Liu and Howell, 2010).

One of the loosely connected modules includes an ER-localized homolog of human RING membrane-anchor E3 ubiquitin ligase RMA1. Due to its cysteine-rich RING finger domain, this gene was incorrectly included in the TPT collection of transcription factors (Coego et al., 2014). In Arabidopsis, *RMA1* forms a small gene family with *RMA2* and *RMA3* (Son et al., 2009). Their tissue expression patterns show certain specificity but all Arabidopsis RMAs show ubiquitin ligase activity at the ER membrane (Matsuda et al., 2001; Son et al., 2009). In human cells, RMA1 ubiquitinates misfolded cystic fibrosis transmembrane conductance regulator (CFTR) at the ER membrane, targeting CFTR for 26S proteasome-mediated degradation (Younger et al., 2006). It is therefore conceivable that Arabidopsis RMA1 may ubiquitinate yet unidentified misfolded proteins at the ER membrane, alleviating the ER stress conditions. A ubiquitination of the unspliced bZIP60 is also an intriguing possibility, as this ER membrane-resident protein might have interesting biological functions (Henriquez-Valencia et al., 2015). In addition, a down-regulation of the ER membrane-localized transcription factor NAC089 confers ER-stress tolerance (Yang et al., 2014b), making it another intriguing candidate for an RMA1-mediated ubiquitination.

The pollen tube growth (PTG) is a process requiring high activity of the secretory system. We queried the co-expression network for an overlap with genes that showed significant upregulation during the PTG (Wang et al., 2008). We identified 19 genes that matched this criterion and most of them were indeed co-expressed with the known UPR signaling machinery (Supplemental Table 4). Interestingly, *MYB109*, a candidate gene identified in our screening, and additional six genes closely co-expressed with *MYB109* were found among these 19 genes, suggesting an involvement of MYB109 transcription factor in the rapid protein synthesis and high physiological activity in the growing pollen tubes.

### Functional characterization of WRKY75 and RMA1 in arabidopsis

We decided to analyze the transgenic lines of WRKY75 (TPT_5.13080.1G) and RMA1 (TPT_4.03510.1I) in more detail. We chose these two genes for the identified strong resistance toward the ER stress-inducing drug in the primary screen and for the localization of the corresponding genes within the co-expression network. WRKY75 was identified within the bZIP60 co-expression cluster, while RMA1 was found only loosely connected with the previously characterized UPR machinery (Figure 3).

**Figure 4:**
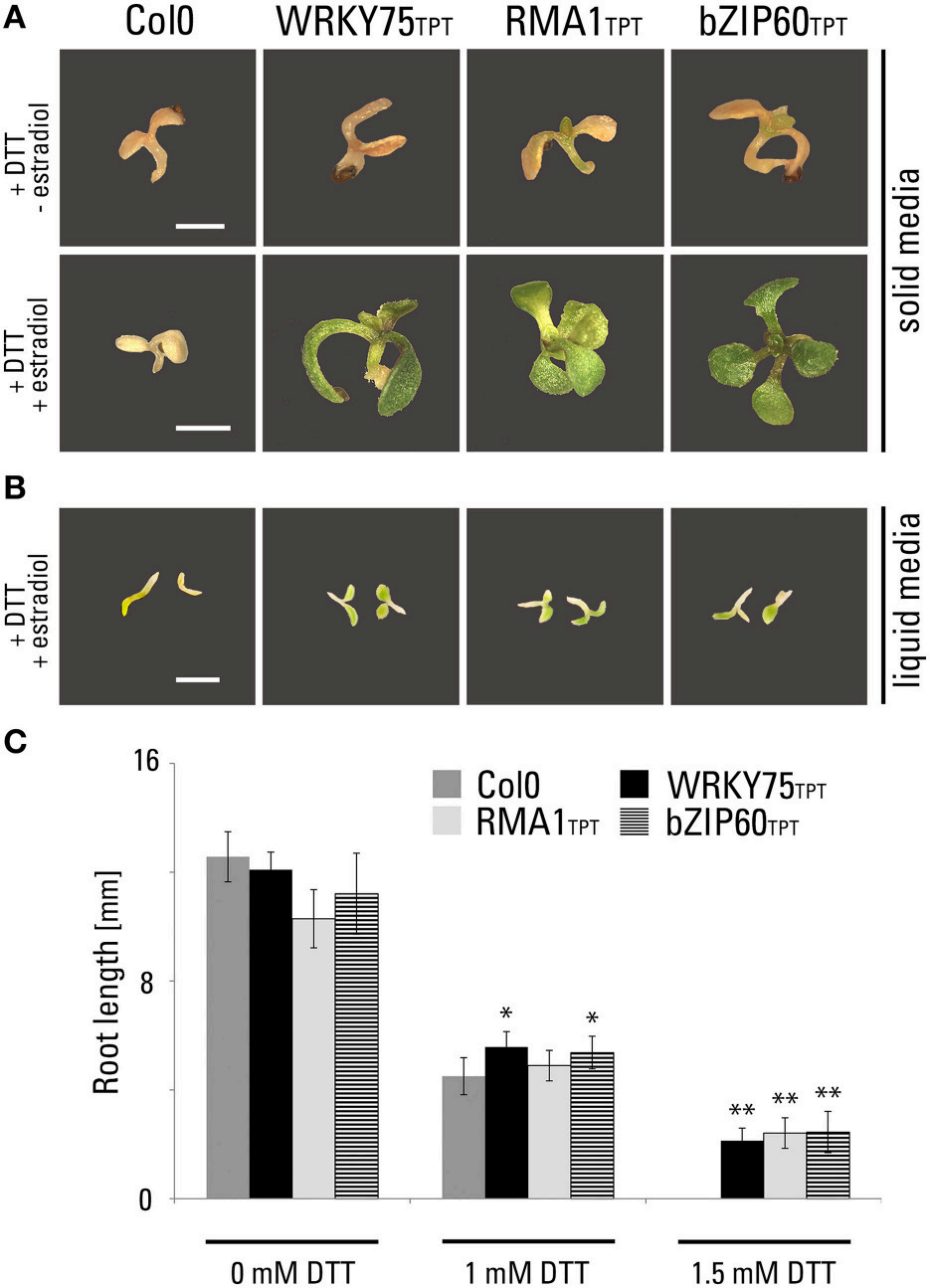
**Phenotypic characterization of inducible transgenic lines expressing WRKY75 and RMA1**. Growth phenotypes of TPT lines of WRKY75 (TPT_5.13080.1G), RMA1 (TPT_4.03510.1I) and bZIP60 (TPT_1.42990.1A) germinated on medium containing DTT. **(A)** Seedlings grown on 1/2 MS agar medium containing 2 mM DTT without estradiol and with 10 μM estradiol for 14 days. **(B)** Seedlings grown in 1/3 MS liquid medium containing 1 mM DTT in the presence of 10 μM estradiol for 5 days at 200 rpm. Scale bar, 2 mm (a, b). **(C)** Root length measurements of vertically grown TPT seedlings. Plants were grown on 1/3 MS agar medium containing different concentration of DTT in the presence of 10 μM estradiol for 5 days. (^*^p < 0.05, ^**^p < 0.01, *t-*Student).

First, we subjected both inducible transgenic lines to DTT, a UPR inducing drug. As a positive control, we used a transgenic line from the TPT collection expressing full-length bZIP60 (TPT_1.42990.1A). All three transgenic lines were found resistant to DTT in both solid (Figure 4A) and liquid (Figure 4B) media. Importantly, this resistance was clearly dependent on the induction of transgene expression by estradiol. Without estradiol, the DTT-treated transgenic seedlings accumulated senescence-associated secondary metabolites (e.g., anthocyanins and polyphenols), while transgenic seedlings treated with estradiol and DTT showed normal green coloration (Figure 4A). We analyzed the root length of seedlings germinated in the presence of DTT and unlike the wild-type control, all three lines showed root growth in the presence of 1.5 mM DTT (Figure 4C).

**Figure 5:**
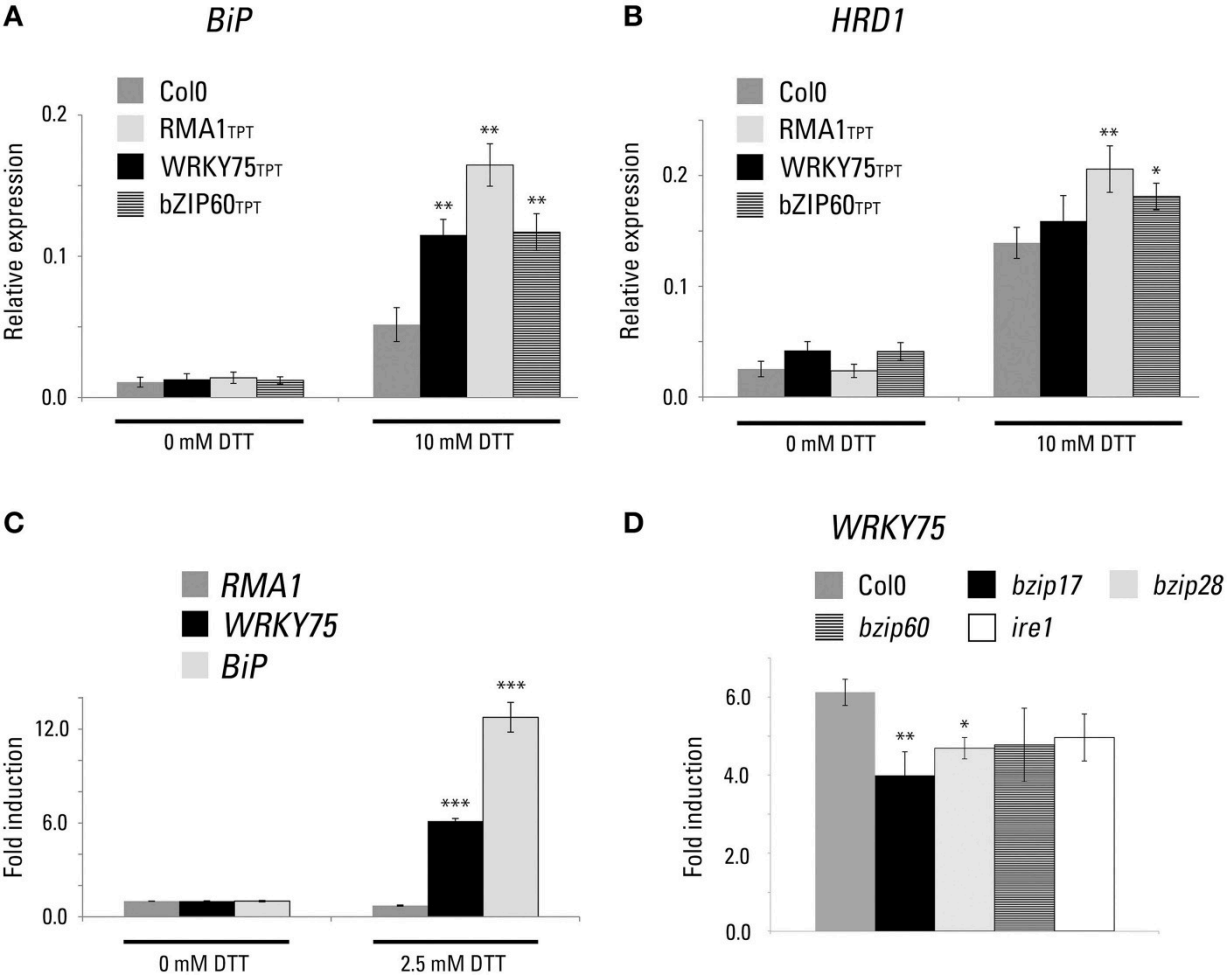
**UPR markers are upregulated in the inducible transgenic lines expressing WRKY75 and RMA1**. WRKY75 is upregulated during ER stress. **(A, B)** Expression of ER-stress markers in the selected TPT lines. The relative expression levels of BiP1/2 **(A)** and HDR1 **(B)** were quantified in response to 10 mM DTT for 5 h in the presence of 10 μM estradiol in the TPT seedlings of WRKY75 (TPT_5.13080.1G), RMA1 (TPT_4.03510.1I), and bZIP60 (TPT_1.42990.1A) seedlings. (^*^p < 0.05, ^**^p < 0.01, *t-*Student) **(C)** Expression of candidate genes in the presence of DTT. The fold induction of RMA1, WRKY75, and BiP3 was quantified in response to 2.5 mM DTT for 5 h in the wild-type Col0 background. (^***^p < 0.001, ANOVA test) **(D)** Expression of WRKY75 in the indicated genetic backgrounds. The fold induction of WRKY75 was quantified in response to 2.5 mM DTT for 5 h. (^**^p < 0.01, ANOVA test).

Subsequently, gene expression response toward the ER-stress inducing drugs was analyzed using previously characterized UPR marker genes. We measured a quantitative expression of *BiP1/2*, the ER-localized chaperons (Blanco-Herrera et al., 2015) and *HRD1*, a homolog of yeast ER membrane-anchored ubiquitin ligase (Kamauchi et al., 2005) using experimental conditions previously described for a genome-wide UPR expression analysis (Martinez and Chrispeels, 2003). In all transgenic lines, we observed a statistically significant upregulation of these marker genes (Figures 5A,B). The strongest transcriptomic response was identified in the RMA1 transgenic line. Taking into consideration that RMA1 is not a transcription factor, the possible explanation for such behavior is the RMA1 ubiquitin ligase activity toward an unknown ER-membrane protein. As a consequence, regulatory mechanisms that control UPR marker expression are being activated.

In continuation, we analyzed a quantitative expression of *RMA1* and *WRKY75* in wild-type Col0 background under conditions that trigger UPR-induced expression changes (Blanco-Herrera et al., 2015). Simultaneously, we analyzed expression of an ER chaperone as a positive control of a DTT-induced gene upregulation. In case of *RMA1*, we did not observe any significant transcriptional response toward DTT. However, *WRKY75* showed significant upregulation triggered by DTT, suggesting a direct participation of this transcription factor in the ER-stress responses (Figure 5C). To dissect a putative participation of WRKY75 in previously described UPR signaling pathways, we analyzed its quantitative expression in various mutant backgrounds. We have not detected substantial differences among analyzed samples, suggesting that WRKY75 expression might be regulated either independently or redundantly of the described UPR signaling pathways (Figure 5D).

### WRKY75 and RMA1 overexpressing lines are more tolerant to salt stress

**Figure 6:**
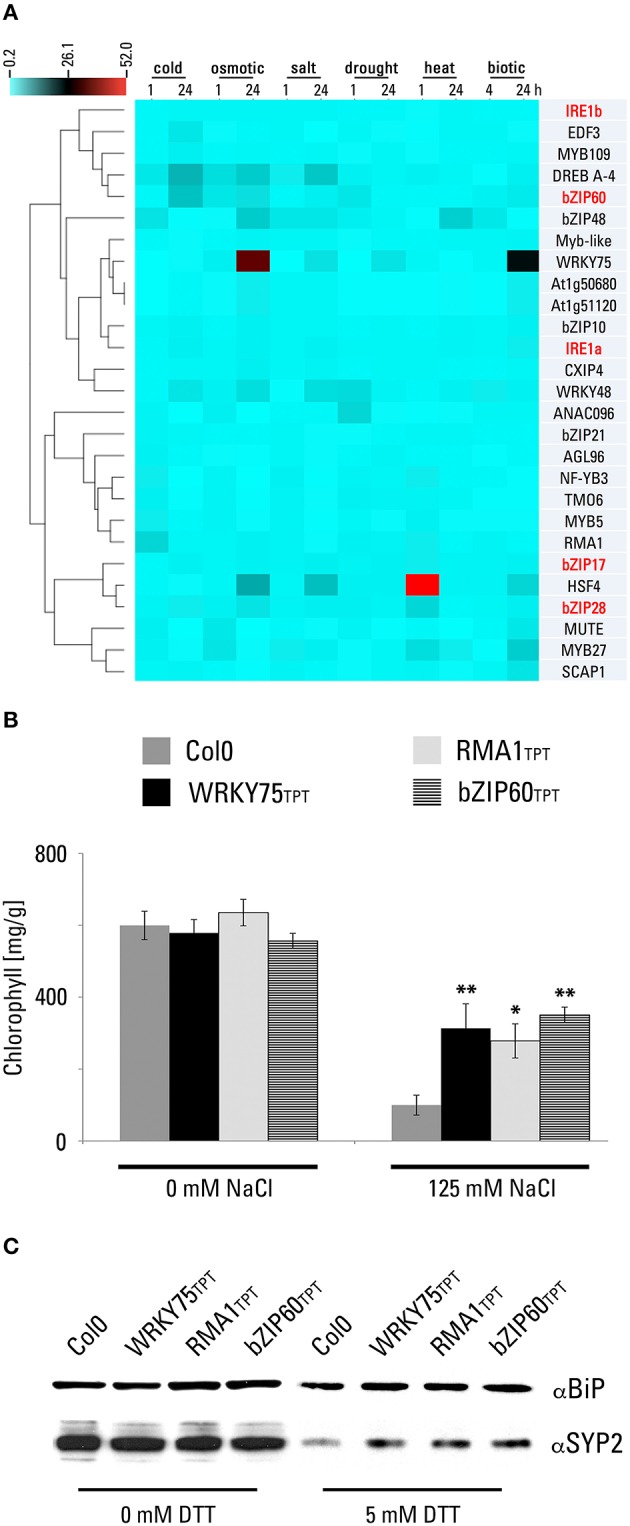
**Inducible lines expressing WRKY75 and RMA1 are more resistant to salt stress. (A)** Digital expression analysis of identified candidate genes and known UPR signaling components (red-typed names) in response to various stress conditions. One-matrix Clustered Image Map was generated using the CIMminer tool with the data listed in Materials and methods. Normalized gene expression levels are displayed on a color-coded scale. **(B)** Salt stress conditions have less impact on selected TPT lines than on wild-type plants. Chlorophyll concentration in methanol extracts of seedlings of transgenic lines expressing WRKY75 (TPT_5.13080.1G), RMA1 (TPT_4.03510.1I), and bZIP60 (TPT_1.42990.1A) was determined as indicated in Materials and Methods. The values represent the average of five biological replicas. (^*^p < 0.05, ^**^p < 0.01, *t-*Student) **(C)** Analysis of endomembrane protein markers BiP and SYP2 in the indicated TPT lines. Four days olds seedlings grown in the liquid MS medium were transferred to the liquid medium supplemented with 10 μM estradiol for 2 days, then treated with 5 mM DTT for 32 h and harvested for total protein extraction.

It was reported that certain components of the UPR signaling also participate in the response to diverse abiotic stresses. In particular, the participation of the UPR machinery in the responses to salt stress was unequivocally demonstrated. While the ER-membrane localized bZIP17 transcription factor was found proteolytically activated by the salt stress conditions (Liu et al., 2007b), its close homolog bZIP28 was not (Liu et al., 2007a). A similar specificity in responses toward salt stress was also observed for the UPR target genes. While the ER-localized BiP3 chaperone was up-regulated by both ER and salt stress, other ER-localized chaperones (calreticulins and protein disulfide isomerases) were not responding to salt stress conditions (Henriquez-Valencia et al., 2015). In addition, the overexpression of bZIP17 lacking the transmembrane domain and the full-length bZIP60 led to salt resistance phenotypes (Fujita et al., 2007; Liu et al., 2008). Furthermore, plants exposed to salt stress show an upregulation in the expression of *bZIP60* (Wang et al., 2011). We decided to analyze transcriptional profiles of all candidate genes under various stress conditions (Figure 6). *RMA1* and *bZIP48* show a rapid upregulation under cold stress, while *DREB A-4* and *bZIP60* present a strong upregulation after a 24-h cold stress treatment. *WRKY75, WRKY48, bZIP48, HSF4*, and *DREB A-4* (At1g77200) were found strongly upregulated after a 24-h osmotic and salt stress treatment. Similarly to *bZIP60, ANAC096*, and *WRKY48* show a rapid upregulation under drought stress. In addition, *HSF4* and *MYB27* manifest a rapid upregulation under heat stress, resembling the *bZIP28* behavior, while *bZIP48* shows an upregulation after a 24-h heat stress treatment. Interestingly, *WRKY75, HSF4*, and *MYB27* show a strong upregulation after inoculation with *P. syringae*, in agreement with previous reports of the UPR signaling machinery association with biotic stress responses (Moreno et al., 2012).

To further characterize *RMA1* and *WRKY75* candidate genes, we were interested whether their overexpression conferred resistance to salt stress in Arabidopsis. As a positive control, we used an inducible transgenic line expressing a full-length *bZIP60* cDNA. To assess the susceptibility/resistance of plants toward salt stress, we analyzed chlorophyll content in Arabidopsis seedlings subjected to 125 mM NaCl for 30 h. In general, we have observed a dramatic decrease in chlorophyll content in treated seedlings compared to the untreated samples (Figure 6B). However, in comparison to the wild type, the transgenic seedlings expressing RMA1, WRKY75, and bZIP60 showed higher chlorophyll content and thus a resistance toward salt stress (Figure 6B).

Similarly to the effects of bZIP17 and bZIP60 overexpression, a manipulation of transcription levels of certain components of the endomembrane trafficking machinery also resulted in resistance to salt stress. In particular, experiments with endosomal Rab7 GTPases in various plant species showed their importance for sequestration of sodium into the vacuole (Mazel et al., 2004; Agarwal et al., 2008). To link the vigor of the endomembrane system to the ER-stress tolerance, we have analyzed the protein levels of the SYP2 endosomal marker in Arabidopsis seedlings subjected to DTT. Analysis of total protein extracts revealed that SYP2 endosomal marker decreased significantly after the DTT treatment (Figure 6C). Nevertheless, in seedlings expressing RMA1, WRKY75, and bZIP60 the rate of decrease was less pronounced than in wild-type plants, suggesting that in these transgenic lines the endosomes were less affected by the ER-stress conditions and likely contributed to the higher salt tolerance. Surprisingly, we have not observed such dramatic decrease in the BiP protein (Figure 6C). We assume that the overexpression of the BiP chaperones during the ER stress caused by the DTT exposure likely compensates the similar changes observed for the SYP2 marker.

## Conclusions and perspectives

In this work, we identified several candidate genes that might participate as effectors in the Unfolded Protein Response. We demonstrated that WRKY75 in particular could be considered a *bona fide* regulator of the ER stress cellular responses. Its expression was found to be directly responding to ER stress-inducing drugs and its overexpression promoted salt stress tolerance. The rest of the candidate genes showed favorable expression patterns in both developmental and abiotic stress transcriptional analyses and therefore can be considered as promising candidates for future studies. We are currently analyzing phenotypical responses of null mutants in several candidate genes toward UPR-inducing drugs.

It is conceivable that manipulating and enhancing the UPR pathways can bring great benefits to plant biotechnology. It has been shown that recombinant protein expression in plant storage tissues is often hampered by the negative consequences of insufficient folding capacities resulting in the ER stress (De Wilde et al., 2013). In addition, certain environmental conditions such as increased temperatures or soil salinity have a dramatic impact on ER folding capacity and as a result on crop productivity. Therefore, identification of novel regulators of the UPR provides a useful tool to design crops with greater tolerance to abiotic and biotic stresses.

## Author contributions

JZ and JV conceived and designed the experiments and performed the bioinformatics analyses. MH, MG, and JZ performed the chemical genomics screening and the subsequent phenotypic characterization. MH and CH performed the qRT-PCR experiments. JZ, JV, JM, and AO wrote the manuscript; all authors contributed to the discussion and approved the final manuscript.

### Conflict of interest statement

The authors declare that the research was conducted in the absence of any commercial or financial relationships that could be construed as a potential conflict of interest.

## References

[B1] Agarwal P. K. Agarwal P. Jain P. Jha B. Reddy M. K. Sopory S. K. (2008). Constitutive overexpression of a stress-inducible small GTP-binding protein PgRab7 from *Pennisetum glaucum* enhances abiotic stress tolerance in transgenic tobacco. Plant Cell Rep. 27, 105–115. 10.1007/s00299-007-0446-0 17899098

[B2] Alonso R. Onate-Sanchez L. Weltmeier F. Ehlert A. Diaz I. Dietrich K.. (2009). A pivotal role of the basic leucine zipper transcription factor bZIP53 in the regulation of Arabidopsis seed maturation gene expression based on heterodimerization and protein complex formation. Plant Cell 21, 1747–1761. 10.1105/tpc.108.062968 19531597 PMC2714925

[B3] Bertolotti A. Wang X. Novoa I. Jungreis R. Schlessinger K. Cho J. H.. (2001). Increased sensitivity to dextran sodium sulfate colitis in IRE1beta-deficient mice. J. Clin. Invest. 107, 585–593. 10.1172/JCI11476 11238559 PMC199427

[B4] Blanco-Herrera F. Moreno A. A. Tapia R. Reyes F. Araya M. D'Alessio C.. (2015). The UDP-glucose: glycoprotein glucosyltransferase (UGGT), a key enzyme in ER quality control, plays a significant role in plant growth as well as biotic and abiotic stress in *Arabidopsis thaliana*. BMC Plant Biol. 15:127. 10.1186/s12870-015-0525-2 26017403 PMC4465474

[B5] Boston R. S. Fontes E. B. Shank B. B. Wrobel R. L. (1991). Increased expression of the maize immunoglobulin binding protein homolog b-70 in three zein regulatory mutants. Plant Cell 3, 497–505. 1840924 10.1105/tpc.3.5.497 PMC160017

[B6] Che P. Bussell J. D. Zhou W. Estavillo G. M. Pogson B. J. Smith S. M. (2010). Signaling from the endoplasmic reticulum activates brassinosteroid signaling and promotes acclimation to stress in Arabidopsis. Sci. Signal 3, ra69. 10.1126/scisignal.2001140 20876872

[B7] Chen Y. Brandizzi F. (2012). AtIRE1A/AtIRE1B and AGB1 independently control two essential unfolded protein response pathways in Arabidopsis. Plant J. 69, 266–277. 10.1111/j.1365-313X.2011.04788.x 21914012

[B8] Coego A. Brizuela E. Castillejo P. Ruiz S. Koncz C. del Pozo J. C.. (2014). The TRANSPLANTA collection of Arabidopsis lines: a resource for functional analysis of transcription factors based on their conditional overexpression. Plant J. 77, 944–953. 10.1111/tpj.12443 24456507

[B9] Conger R. Chen Y. Fornaciari S. Faso C. Held M. A. Renna L.. (2011). Evidence for the involvement of the Arabidopsis SEC24A in male transmission. J. Exp. Bot. 62, 4917–4926. 10.1093/jxb/err174 21705385 PMC3193003

[B10] da Silva Conceicao A. Marty-Mazars D. Bassham D. C. Sanderfoot A. A. Marty F. Raikhel N. V. (1997). The syntaxin homolog AtPEP12p resides on a late post-Golgi compartment in plants. Plant Cell 9, 571–582. 9144962 PMC156940

[B11] Deng Y. Humbert S. Liu J. X. Srivastava R. Rothstein S. J. Howell S. H. (2011). Heat induces the splicing by IRE1 of a mRNA encoding a transcription factor involved in the unfolded protein response in Arabidopsis. Proc. Natl. Acad. Sci. U.S.A. 108, 7247–7252. 10.1073/pnas.1102117108 21482766 PMC3084119

[B12] De Wilde K. De Buck S. Vanneste K. Depicker A. (2013). Recombinant antibody production in Arabidopsis seeds triggers an unfolded protein response. Plant Physiol. 161, 1021–1033. 10.1104/pp.112.209718 23188806 PMC3561000

[B13] Fujita M. Mizukado S. Fujita Y. Ichikawa T. Nakazawa M. Seki M.. (2007). Identification of stress-tolerance-related transcription-factor genes via mini-scale Full-length cDNA Over-eXpressor (FOX) gene hunting system. Biochem. Biophys. Res. Commun. 364, 250–257. 10.1016/j.bbrc.2007.09.124 17937930

[B14] Gao H. Brandizzi F. Benning C. Larkin R. M. (2008). A membrane-tethered transcription factor defines a branch of the heat stress response in *Arabidopsis thaliana*. Proc. Natl. Acad. Sci. U.S.A. 105, 16398–16403. 10.1073/pnas.0808463105 18849477 PMC2571009

[B15] Harding H. P. Zhang Y. Ron D. (1999). Protein translation and folding are coupled by an endoplasmic-reticulum-resident kinase. Nature 397, 271–274. 10.1038/16729 9930704

[B16] Hatano K. Shimada T. Hiraiwa N. Nishimura M. Hara-Nishimura I. (1997). A rapid increase in the level of binding protein (BiP) is accompanied by synthesis and degradation of storage proteins in pumpkin cotyledons. Plant Cell Physiol. 38, 344–351. 9150606 10.1093/oxfordjournals.pcp.a029172

[B17] Haze K. Yoshida H. Yanagi H. Yura T. Mori K. (1999). Mammalian transcription factor ATF6 is synthesized as a transmembrane protein and activated by proteolysis in response to endoplasmic reticulum stress. Mol. Biol. Cell 10, 3787–3799. 10564271 10.1091/mbc.10.11.3787 PMC25679

[B18] Henriquez-Valencia C. Moreno A. A. Sandoval-Ibanez O. Mitina I. Blanco-Herrera F. Cifuentes-Esquivel N.. (2015). bZIP17 and bZIP60 regulate the expression of BiP3 and other salt stress responsive genes in an UPR-independent manner in *Arabidopsis thaliana*. J. Cell. Biochem. 116, 1638–1645. 10.1002/jcb.25121 25704669

[B19] Howell S. H. (2013). Endoplasmic reticulum stress responses in plants. Annu. Rev. Plant Biol. 64, 477–499. 10.1146/annurev-arplant-050312-120053. 23330794

[B20] Ishiguro S. Watanabe Y. Ito N. Nonaka H. Takeda N. Sakai T.. (2002). SHEPHERD is the Arabidopsis GRP94 responsible for the formation of functional CLAVATA proteins. EMBO J. 21, 898–908. 10.1093/emboj/21.5.898 11867518 PMC125899

[B21] Iwata Y. Fedoroff N. V. Koizumi N. (2008). Arabidopsis bZIP60 is a proteolysis-activated transcription factor involved in the endoplasmic reticulum stress response. Plant Cell 20, 3107–3121. 10.1105/tpc.108.061002 19017746 PMC2613661

[B22] Kamauchi S. Nakatani H. Nakano C. Urade R. (2005). Gene expression in response to endoplasmic reticulum stress in *Arabidopsis thaliana*. FEBS J. 272, 3461–3476. 10.1111/j.1742-4658.2005.04770.x 15978049

[B23] Kilian J. Whitehead D. Horak J. Wanke D. Weinl S. Batistic O.. (2007). The AtGenExpress global stress expression data set: protocols, evaluation and model data analysis of UV-B light, drought and cold stress responses. Plant J. 50, 347–363. 10.1111/j.1365-313X.2007.03052.x 17376166

[B24] Koiwa H. Li F. McCully M. G. Mendoza I. Koizumi N. Manabe Y.. (2003). The STT3a subunit isoform of the Arabidopsis oligosaccharyltransferase controls adaptive responses to salt/osmotic stress. Plant Cell 15, 2273–2284. 10.1105/tpc.013862 12972670 PMC197294

[B25] Koizumi N. Martinez I. M. Kimata Y. Kohno K. Sano H. Chrispeels M. J. (2001). Molecular characterization of two Arabidopsis Ire1 homologs, endoplasmic reticulum-located transmembrane protein kinases. Plant Physiol. 127, 949–962. 10.1104/pp.010636 11706177 PMC129266

[B26] Liu J. X. Howell S. H. (2010). bZIP28 and NF-Y transcription factors are activated by ER stress and assemble into a transcriptional complex to regulate stress response genes in Arabidopsis. Plant Cell 22, 782–796. 10.1105/tpc.109.072173 20207753 PMC2861475

[B27] Liu J. X. Srivastava R. Che P. Howell S. H. (2007a). An endoplasmic reticulum stress response in Arabidopsis is mediated by proteolytic processing and nuclear relocation of a membrane-associated transcription factor, bZIP28. Plant Cell 19, 4111–4119. 10.1105/tpc.106.050021 18156219 PMC2217655

[B28] Liu J. X. Srivastava R. Che P. Howell S. H. (2007b). Salt stress responses in Arabidopsis utilize a signal transduction pathway related to endoplasmic reticulum stress signaling. Plant J. 51, 897–909. 10.1111/j.1365-313X.2007.03195.x 17662035 PMC2156172

[B29] Liu J. X. Srivastava R. Howell S. H. (2008). Stress-induced expression of an activated form of AtbZIP17 provides protection from salt stress in Arabidopsis. Plant Cell Environ. 31, 1735–1743. 10.1111/j.1365-3040.2008.01873.x 18721266

[B30] Liu Y. Burgos J. S. Deng Y. Srivastava R. Howell S. H. Bassham D. C. (2012). Degradation of the endoplasmic reticulum by autophagy during endoplasmic reticulum stress in Arabidopsis. Plant Cell 24, 4635–4651. 10.1105/tpc.112.101535 23175745 PMC3531857

[B31] Martinez I. M. Chrispeels M. J. (2003). Genomic analysis of the unfolded protein response in Arabidopsis shows its connection to important cellular processes. Plant Cell 15, 561–576. 10.1105/tpc.007609 12566592 PMC141221

[B32] Matsuda N. Suzuki T. Tanaka K. Nakano A. (2001). Rma1, a novel type of RING finger protein conserved from Arabidopsis to human, is a membrane-bound ubiquitin ligase. J. Cell Sci. 114, 1949–1957. 11329381 10.1242/jcs.114.10.1949

[B33] Mazel A. Leshem Y. Tiwari B. S. Levine A. (2004). Induction of salt and osmotic stress tolerance by overexpression of an intracellular vesicle trafficking protein AtRab7 (AtRabG3e). Plant Physiol. 134, 118–128. 10.1104/pp.103.025379 14657401 PMC316292

[B34] Moreno A. A. Mukhtar M. S. Blanco F. Boatwright J. L. Moreno I. Jordan M. R.. (2012). IRE1/bZIP60-mediated unfolded protein response plays distinct roles in plant immunity and abiotic stress responses. PLoS ONE 7:e31944. 10.1371/journal.pone.0031944 22359644 PMC3281089

[B35] Mori K. Kawahara T. Yoshida H. Yanagi H. Yura T. (1996). Signalling from endoplasmic reticulum to nucleus: transcription factor with a basic-leucine zipper motif is required for the unfolded protein-response pathway. Genes Cells 1, 803–817. 9077435 10.1046/j.1365-2443.1996.d01-274.x

[B36] Nagashima Y. Iwata Y. Ashida M. Mishiba K. Koizumi N. (2014). Exogenous salicylic acid activates two signaling arms of the unfolded protein response in Arabidopsis. Plant Cell Physiol. 55, 1772–1778. 10.1093/pcp/pcu108 25138441

[B37] Obayashi T. Hayashi S. Saeki M. Ohta H. Kinoshita K. (2009). ATTED-II provides coexpressed gene networks for Arabidopsis. Nucleic Acids Res. 37, D987–D991. 10.1093/nar/gkn807 18953027 PMC2686564

[B38] Ogata M. Hino S. Saito A. Morikawa K. Kondo S. Kanemoto S.. (2006). Autophagy is activated for cell survival after endoplasmic reticulum stress. Mol. Cell. Biol. 26, 9220–9231. 10.1128/MCB.01453-06 17030611 PMC1698520

[B39] Pajerowska-Mukhtar K. M. Wang W. Tada Y. Oka N. Tucker C. L. Fonseca J. P.. (2012). The HSF-like transcription factor TBF1 is a major molecular switch for plant growth-to-defense transition. Curr. Biol. 22, 103–112. 10.1016/j.cub.2011.12.015 22244999 PMC3298764

[B40] Porra R. J. Thompson W. A. Kriedemann P. E. (1989). Determination of accurate extinction coefficients and simultaneous equations for assaying chlorophylls a and b extracted with four different solvents: verification of the concentration of chlorophyll standards by atomic absorption spectroscopy. Biochim. Biophys. Acta Bioenerg. 975, 384–394. 10.1016/S0005-2728(89)80347-0

[B41] Prischi F. Nowak P. R. Carrara M. Ali M. M. (2014). Phosphoregulation of Ire1 RNase splicing activity. Nat. Commun. 5, 3554. 10.1038/ncomms4554 24704861 PMC3988810

[B42] Schmid M. Davison T. S. Henz S. R. Pape U. J. Demar M. Vingron M.. (2005). A gene expression map of *Arabidopsis thaliana* development. Nat. Genet. 37, 501–506. 10.1038/ng1543 15806101

[B43] Shannon P. Markiel A. Ozier O. Baliga N. S. Wang J. T. Ramage D.. (2003). Cytoscape: a software environment for integrated models of biomolecular interaction networks. Genome Res. 13, 2498–2504. 10.1101/gr.1239303 14597658 PMC403769

[B44] Sidrauski C. Walter P. (1997). The transmembrane kinase Ire1p is a site-specific endonuclease that initiates mRNA splicing in the unfolded protein response. Cell 90, 1031–1039. 9323131 10.1016/s0092-8674(00)80369-4

[B45] Siefers N. Dang K. K. Kumimoto R. W. Bynum W. E. Tayrose G. Holt B. F. III. (2009). Tissue-specific expression patterns of Arabidopsis NF-Y transcription factors suggest potential for extensive combinatorial complexity. Plant Physiol. 149, 625–641. 10.1104/pp.108.130591 19019982 PMC2633833

[B46] Son O. Cho S. K. Kim E. Y. Kim W. T. (2009). Characterization of three Arabidopsis homologs of human RING membrane anchor E3 ubiquitin ligase. Plant Cell Rep. 28, 561–569. 10.1007/s00299-009-0680-8 19224217

[B47] Srivastava R. Chen Y. Deng Y. Brandizzi F. Howell S. H. (2012). Elements proximal to and within the transmembrane domain mediate the organelle-to-organelle movement of bZIP28 under ER stress conditions. Plant J. 70, 1033–1042. 10.1111/j.1365-313X.2012.04943.x 22335396

[B48] Sun L. Yang Z. T. Song Z. T. Wang M. J. Sun L. Lu S. J.. (2013). The plant-specific transcription factor gene *NAC103* is induced by bZIP60 through a new cis-regulatory element to modulate the unfolded protein response in Arabidopsis. Plant J. 76, 274–286. 10.1111/tpj.12287 23869562

[B49] Tirasophon W. Welihinda A. A. Kaufman R. J. (1998). A stress response pathway from the endoplasmic reticulum to the nucleus requires a novel bifunctional protein kinase/endoribonuclease (Ire1p) in mammalian cells. Genes Dev. 12, 1812–1824. 9637683 10.1101/gad.12.12.1812 PMC316900

[B50] Toufighi K. Brady S. M. Austin R. Ly E. Provart N. J. (2005). The botany array resource: e-northerns, expression angling, and promoter analyses. Plant J. 43, 153–163. 10.1111/j.1365-313X.2005.02437.x 15960624

[B51] Walter P. Ron D. (2011). The unfolded protein response: from stress pathway to homeostatic regulation. Science 334, 1081–1086. 10.1126/science.1209038 22116877

[B52] Wang M. Xu Q. Yuan M. (2011). The unfolded protein response induced by salt stress in Arabidopsis. Methods Enzymol. 489, 319–328. 10.1016/B978-0-12-385116-1.00018-2 21266238

[B53] Wang X. Z. Harding H. P. Zhang Y. Jolicoeur E. M. Kuroda M. Ron D. (1998). Cloning of mammalian Ire1 reveals diversity in the ER stress responses. EMBO J. 17, 5708–5717. 10.1093/emboj/17.19.5708 9755171 PMC1170899

[B54] Wang Y. Zhang W. Z. Song L. F. Zou J. J. Su Z. Wu W. H. (2008). Transcriptome analyses show changes in gene expression to accompany pollen germination and tube growth in Arabidopsis. Plant Physiol. 148, 1201–1211. 10.1104/pp.108.126375 18775970 PMC2577266

[B55] Winter D. Vinegar B. Nahal H. Ammar R. Wilson G. V. Provart N. J. (2007). An “Electronic Fluorescent Pictograph” browser for exploring and analyzing large-scale biological data sets. PLoS ONE 2:e718. 10.1371/journal.pone.0000718 17684564 PMC1934936

[B56] Yang K. Z. Xia C. Liu X. L. Dou X. Y. Wang W. Chen L. Q.. (2009). A mutation in Thermosensitive male sterile 1, encoding a heat shock protein with DnaJ and PDI domains, leads to thermosensitive gametophytic male sterility in Arabidopsis. Plant J. 57, 870–882. 10.1111/j.1365-313X.2008.03732.x 18980646

[B57] Yang Z. T. Lu S. J. Wang M. J. Bi D. L. Sun L. Zhou S. F.. (2014a). A plasma membrane-tethered transcription factor, NAC062/ANAC062/NTL6, mediates the unfolded protein response in Arabidopsis. Plant J. 79, 1033–1043. 10.1111/tpj.12604 24961665

[B58] Yang Z. T. Wang M. J. Sun L. Lu S. J. Bi D. L. Sun L.. (2014b). The membrane-associated transcription factor NAC089 controls ER-stress-induced programmed cell death in plants. PLoS Genet. 10:e1004243. 10.1371/journal.pgen.1004243 24675811 PMC3967986

[B59] Yoshida H. Matsui T. Yamamoto A. Okada T. Mori K. (2001). XBP1 mRNA is induced by ATF6 and spliced by IRE1 in response to ER stress to produce a highly active transcription factor. Cell 107, 881–891. 10.1016/S0092-8674(01)00611-0 11779464

[B60] Younger J. M. Chen L. Ren H. Y. Rosser M. F. Turnbull E. L. Fan C. Y.. (2006). Sequential quality-control checkpoints triage misfolded cystic fibrosis transmembrane conductance regulator. Cell 126, 571–582. 10.1016/j.cell.2006.06.041 16901789

